# Hybrid ultrasound and landmark technique for thoracic paravertebral block: a clinical image

**DOI:** 10.1016/j.bjane.2024.844555

**Published:** 2024-09-06

**Authors:** Anthony M.-H. Ho, Glenio B. Mizubuti, Gregory Klar, Rachel Rooney

**Affiliations:** Queen's University, Kingston General Hospital, Department of Anesthesiology and Perioperative Medicine, Kingston, Ontario, Canada

Thoracic Paravertebral Block (TPVB) remains the gold standard for unilateral thoracic block and provides more reliable analgesia than the Erector Spinae Plane Block (ESPB).

Ultrasound (US)-guided TPVB can be challenging even for experienced US-trained regional anesthesiologists due to the depth of the paravertebral space and the steep angle of the block needle, especially in overweight patients. In one study in non-obese adult patients (n = 39; weight 67 ± 7.4 kg), the first needle pass/attempt success rate was 76.9%, requiring multiple attempts.^1^ In comparison, the landmark technique is simple and requires no US skills. It involves landing the block needle tip on the transverse process (TP), walking the needle over/under the TP, and advancing it 1 cm.^2^

The challenge of the landmark technique, however, is finding the TP. The location of the TP in relation to the intervertebral space varies with body habitus, thoracic level, and spine curvature. The depth of the TP also varies considerably. If the bone is not reached at a certain estimated depth, the needle is either too shallow or in between TPs, and its angle and/or entry site must be adjusted to ensure “safe landing” on the TP and avoid pleural violation. Therefore, knowing the location and depth of the TP virtually eliminates the risk of the needle being advanced between TPs, and reduces the risk of the needle remaining too shallow (missing the TP) or going too deep [risking a pneumothorax (incidence of 0.5%)],^3^ thereby improving efficiency and patient safety/comfort. Hence, we suggest performing a scout US scan to identify the desired TP and its depth, which requires minimal US skill (Fig. 1). A high-frequency linear US probe in the sagittal plane 2.5 cm (in most adults) lateral to midline is usually adequate, although a low-frequency curvilinear probe may occasionally be required, particularly in morbidly obese patients. If the superior costotransverse ligament (and pleura) is visualized, the distance beyond the TP can also be noted (with provision for the change in the needle angle). The TPVB can then be completed by walking the needle over/under the TP and advancing it for 1 cm, or until a subtle loss of resistance is felt.^2^ Contraindications for this hybrid technique do not differ from other TPVB approaches and are beyond the scope of this article. Anecdotally, we have used this technique for several years with no observed complications (e.g., pneumothorax) and arguably shorter procedural time when compared to the US-guided technique, especially for technically challenging blocks. This technique relies almost entirely on tactile feeling and is particularly suitable for anesthesiologists without formal US training and who had been reluctant to perform TPVB because of the challenge of finding and “safe landing” on the TP. It is also an excellent backup technique when US-guided TPVB proves difficult/impossible. A case in point was that of a morbidly obese patient with rib fractures in whom multiple attempts by two experienced regional anesthesiologists and a competent final-year resident at establishing an US-guided ESPB had failed (could see the TP but not the needle tip); eventually, a TPVB was established using this hybrid technique. Our next step is to start teaching this hybrid technique both locally and in an upcoming foreign mission, and to report our experience. We expect the learning curve of this technique to be easier than both the traditional landmark and the US-guided technique.

**Figure 1:**
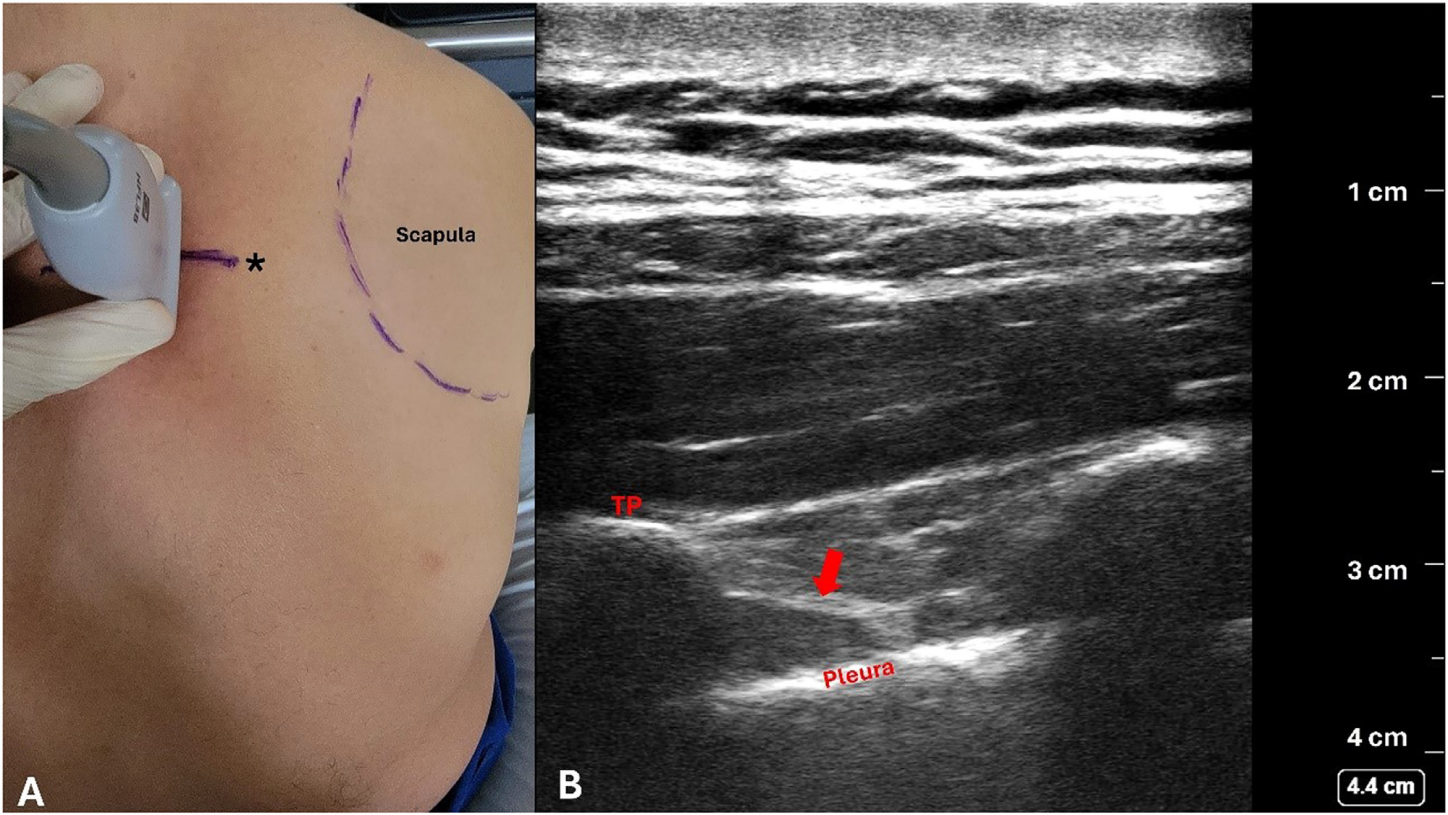
Hybrid (ultrasound and landmark) approach to thoracic paravertebral block. Upon initial scanning, a skin mark (*) is made to indicate the center of the transverse process (TP) under the ultrasound probe (A). The pressure of the probe on the patient's back is then eased to note the TP's actual depth – in this case, approximately 2.5 cm (B). Knowing the location and the depth of the TP facilitates achieving the “safe landing” of the block needle with the landmark technique. The needle can then be walked off the TP and advanced approximately 1 cm, or until a subtle loss of resistance is felt upon passing through the superior costotransverse ligament (red arrow) – in this case, at approximately 3 cm deep (B). In the picture, the male subject is one of the co-authors who is 72 kg and 1.65 m (body mass index 26 kg.m^-2^).

## Conflicts of interest

The authors declare no conflicts of interest.
